# Supplementary material for: Characterization of the bacterial fecal microbiota composition of pigs preceding the clinical signs of swine dysentery
Source: PLoS One. 2023 Nov 10;18(11):e0294273. doi: 10.1371/journal.pone.0294273 (PMC10637667; doi:10.1371/journal.pone.0294273)
Supplement: S1 Table — (PDF) [file pone.0294273.s001.pdf]

S1 Table. Ingredients and nutrient specs of swine dysentery custom diet (JH002_CFRC)

**Ingredient**	**%**	**Nutrient**	**%**
Barley	0	Crude protein	21.1
Wheat (hard, spring)	42.24	Crude Fiber	5.9
Corn DDGS	25	Crude Fat (ether extract)	6.42
Soybean meal (50%)	4.17	Digestible Energy (NE)	2,400 Kcal
Canola meal (40%)	16.46	Calcium (total)**	0.21
Wheat bran	7.5	Phosphorus (total)	0.61
Calcium carbonate**	0	Sodium	0.28
Canola oil	3.5	Lysine (SID)	0.95
Salt (NaCl)	0.3	Methionine (SID)	0.32
Lysine	0.4	Threonine (SID)	0.59
Phytase	0.02	Tryptophan (SID)	0.19
Micro	0.4	Met + Cys (SID)	0.72
**Total**	**100**	Acid detergent fiber	7.24
		Neutral detergent fiber	22.85
		Cellulose	9.85
		Lignin	2.43
		Copper (ppm)	37.6
		Zinc (ppm)	179

* Manufactured with 10/64 screen with a target particle size of 700 microns
** inadvertent error with limestone omitted
NE=net energy, SID=standardized ileal digestibility
