# Supplementary material for: Characterization of the bacterial fecal microbiota composition of pigs preceding the clinical signs of swine dysentery
Source: PLoS One. 2023 Nov 10;18(11):e0294273. doi: 10.1371/journal.pone.0294273 (PMC10637667; doi:10.1371/journal.pone.0294273)
Supplement: S2 Table — (PDF) [file pone.0294273.s002.pdf]

Table S2. Description of fecal samples used in this study.

Pig	Pen	Sampling day (DPI)[1]			
		**d0**[2]	**d-2SD**[3]	**d-1SD**[4]	**d-MHD**[5]
358	Pen 1	0	7	8	9
385	Pen 1	0	7	8	9
484	Pen 1	0	10	11	12
359	Pen 1	0	13	14	15
368	Pen 2	0	9	10	15
573	Pen 3	0	16	17	18
370	Pen 3	0	12	13	14
374	Pen 3	0	7	8	9
410	Pen 3	0	12	13	14
387	Pen 8	0	7	8	9
381	Pen 8	0	7	8	9
392	Pen 8	0	13	14	15
435	Pen 9	0	5	6	7
576	Pen 9	0	7	8	9
593	Pen 10	0	12	13	14

[1]DPI: days post introduction of seeder pigs, [2]d0: day after contact with seeder pigs (n=15), [3]d-2SD: 2 days before mucohaemorrhagic diarrhea was observed (n=15), [4]d-1SD: one day before mucohaemorrhagic diarrhea was observed (n=15), [5]MHD: day mucohaemorrhagic diarrhea was observed for the first time (n=14).
