# Supplementary material for: Characterization of the bacterial fecal microbiota composition of pigs preceding the clinical signs of swine dysentery
Source: PLoS One. 2023 Nov 10;18(11):e0294273. doi: 10.1371/journal.pone.0294273 (PMC10637667; doi:10.1371/journal.pone.0294273)
Supplement: S3 Table — (PDF) [file pone.0294273.s003.pdf]

Table S3. Amplicon sequence variants (ASV) with significant differential abundance between d0 and all other days.

	ASV	Total read counts				logFC[5]	adjusted *P*-value
		**d0**[1]	**d-2SD**[2]	**d-1SD**[3]	**MHD**[4]		
d0 vs. d-2SD	*Alistipes sp. JC136*	8154	4867	10578	9037	1.914	0.022
	Acetanaerobacterium elongatum	7961	4030	7571	4520	2.386	0.000
	Limosilactobacillus frumenti	592	5606	3661	4042	-2.276	0.033
d0 vs. d-1SD	*Prevotella baroniae*	8918	8306	8530	11078	1.341	0.024
	Dialister succinatiphilus	13411	4355	4583	1866	1.968	0,015
	Limosilactobacillus frumenti	592	5606	3661	4042	-2.463	0.025
d0 vs. d-MHD	*Phascolarctobacterium sp.*	51619	79540	83848	105144	-1.037	0.024
	Cohnella fermenti	11475	19674	25019	14247	0,610	0.030
	Barnesiella intestinihominis	11117	10148	17669	9807	0.957	0.030
	Parabacteroides gordonii	4891	6228	10456	17377	-1.266	0.008
	Eubacterium brachy	10230	7112	7813	1546	3.324	0.000
	Selenomonas bovis	5056	7415	9064	5154	1.072	0.018
	Dialister succinatiphilus	13411	4355	4583	1866	2.705	0.000
	Acetanaerobacterium elongatum	7961	4030	7571	4520	1.758	0.007
	Flavonifractor sp.	2203	4221	5639	8325	-1.583	0.002

Ruminococcus callidus	6150	3611	5500	1977	1.953	0.033
Prevotella buccae	2454	1967	3943	6224	-2.096	0.030
Alistipes dispar	1321	1789	3089	8254	-2.532	0.024
Limosilactobacillus frumenti	592	5606	3661	4042	-2.279	0.014
Pygmaiobacter massiliensis	4084	2929	3860	2889	1.323	0.005
Ammonifex degensii	1024	3047	4653	4723	-1.865	0.007

[1]d0: day after contact with seeder pigs (n=15), [2]d-2SD: 2 days before mucohaemorrhagic diarrhea was observed (n=15), [3]d-1SD: one day before mucohaemorrhagic diarrhea was observed (n=15), [4]MHD: day mucohaemorrhagic diarrhea was observed for the first time (n=14).[5]The degree of differential abundance is represented by log_2 fold change (logFC) between d0 and all other sample days.
