# Supplementary material for: Characterization of the bacterial fecal microbiota composition of pigs preceding the clinical signs of swine dysentery
Source: PLoS One. 2023 Nov 10;18(11):e0294273. doi: 10.1371/journal.pone.0294273 (PMC10637667; doi:10.1371/journal.pone.0294273)
Supplement: Attachment — Submitted filename: response.docx [file pone.0294273.s005.docx]

The authors addressed the reviewers' comments satisfactorily. Below are some of my minor comments preceding acceptance.
Abstract:
I’m not sure that readers can understand what the authors intend for “seeder pig” and “contact pigs” reading only abstract section.

A: Addressed.
The abstract can’t finish with this statement “Future investigations to verify the specific role of these taxa on the pathogenesis of SD is warranted” but requires the implications and the limitations of this study in details.

A: Updated.
Introduction:
Line 40: Delete “initial”

A: Deleted.
Lines 43-49: This list of studies should be preceded by an introductory sentence such as "Numerous studies in the literature have demonstrated the role etc."

A: Updated.
Line 56: italics?

A: Phyla names do not need to be itacilized.
Line 63: I advise the authors to reverse "colonic microbiome" and "incidence of SD".

A: Updated.
Line 73-75: Change this sentence as follows: We assessed any disturbances in the fecal microbiome immediately before clinical SD was observed.
Authors should specify what they consider “immediately prior to the clinical SD”. Was it during incubation? How many days?

A: Addressed (L75).
Discussion:
Line 225: Please change “stepping-stone” in something else.

A: Changed to “intermediary step”/
What are the implications of this study? Understanding the microbiome in order to intervene in some way?
The limitation of the few samples analyzed was discussed, not the fact that many parameters would not seem to differ between infected and non-infected animals.

A: We refrained from overinterpreting the data. Microbiome-host-pathogen interactions are extremely complex and cannot be fully dissected with a single study looking at the bacteriome. We have addressed this issue in lines 306-307.

It is unclear what the editor means by “many parameters”? If this is related to alpha/beta diversity, that is expected given the short amount of time between sample collection points. All studies published to date looking at microbiome changes due to SD sampled animals at peak clinical signs vs before inoculation, when lesions and disease are well underway (which takes 7-14 days). In the case of our study, the very short period between d-2 and clinical disease (MHD, 48h) likely contributed to the lack of differences between parameters – which was expected. In addition, the editor may want to revise the methodology employed here: there is no infected vs non-infected comparison in this study.
