# Supplementary material for: Characterization of the bacterial fecal microbiota composition of pigs preceding the clinical signs of swine dysentery
Source: PLoS One. 2023 Nov 10;18(11):e0294273. doi: 10.1371/journal.pone.0294273 (PMC10637667; doi:10.1371/journal.pone.0294273)
Supplement: Attachment — Submitted filename: Responses to reviewer comments.docx [file pone.0294273.s004.docx]

**We appreciate the reviewer’s unpaid time and efforts in reviewing this manuscript. Thank you!**

**Reviewer #1:** The manuscript is designed to characterize the intestinal microbiota of piglets prior to inoculum with the bacterium that causes swine dysentery. , and contrast the findings with the profiles of the microbiota of pigs exposed 1 or 2 days before the presence of hemorrhagic diarrhea.

1- I want to understand that the purpose is to define if there is a bacterial profile in these pigs that is permissive to develop hemorrhagic dysenteria. However, the purpose is not explicitly clear because the methodology does not mention a study design as such, on the other hand , although the hypothesis of the authors is mentioned.

A: The purpose of the study is to characterize changes in the fecal microbiome immediately prior to disease. Previous studies have only characterized the changes in the microbiome of clinically affected pigs versus healthy controls or samples collected prior to inoculation (See change in L54). As stated in L74 (where a hypothesis and goal are described), we aimed to profile the changes in fecal microbiome composition immediately prior to the development of SD.

2- The methodology section is mixed with what would be part of the results and in the section where the description of the results should be, the figures are described as the figure captions were. Thus, the manuscript does not conform to the standards of the journal.

A: It is unclear to us as what the reviewer perceived as “methodology section is mixed with results”. Unfortunately, the Reviewer did not provide specifics, so we are unable to address this issue. That being said, we believe that they might refer to Table 1, which is truly part of the methods rather than results. Sampling (and the “result” from inoculation) is a key aspect of the methodology used in this study since we required a prior knowledge of clinical outcomes in order to analyze the correct samples (1 and 2 days prior to SD).

PLOS One guidelines to authors states that “Each figure caption should appear directly after the paragraph in which they are first cited.”. The Reviewer can familiarize themselves with these guidelines by clicking here: <https://journals.plos.org/plosone/s/submission-guidelines>

3- Reading the introduction section, I was struck by the fact that the authors mention that there is evidence on this same model, with results comparable to those presented in this manuscript. In my opinion, the manuscript should be structured in a consistent way, making it clear: 1- what is the purpose and the novelty of this research strategy with respect to what has already been published;

A: We regret that the introduction was not clearly written, leading to some confusion by the reviewer. We have modified the text (L54 and L73) to hopefully clarify it. Our results differ from other published work from the perspective that those studies did NOT sample pigs immediately prior to the development of disease. Pigs were sampled prior to inoculation, and not naturally infect but inoculated directly with an overload of pathogen. In our case, we analyzed samples from pigs that were naturally infected with Brachyspira, but were still in the incubation period (text modified in L77).

2- the methodology section describe the methodology or, where appropriate, 3- gather methodology and results in a single section.

A: We hope to have clarified this in the comments above*.*

4- discuss the results and contrast them with those obtained by other authors, mention the limitations of the model and highlight the most important findings in a conclusion section.

A: Thank you, this can be found in L223-300.

**Reviewer #2: General comments:**

This is very well written manuscript that has a clear study design. The main rationale for the study was to understand the change in the fecal microbiota few days before the development of mucohemorrhagic diarrhea due to swine dysentery in pigs. For the study, they used a seeder exposure method that it is interesting to mimic natural infection. Despite the difference in the days until the animals started with mucohemorrhagic diarrhea, fecal samples were collected daily, demonstrating the complexity of the study design. Chao1 Index between d-1SD and MHD compared to D0 was demonstrated as the only change in alpha diversity.

Alistipes dispar and Parabacterioides gordonii increased in MHD sound as an interesting finding that may help to elucidate *B. hyodysenteriae* pathogenesis. It would be interesting to see some discussion about why *P. gordonii* has been observed in health pigs but had demonstrated a 3-fold increase in diseased animals (MHD).

A: Acknowledged. We have added more info on P. gordonii in L230-240. Unfortunately, there’s nothing very clear regarding how this bacterium could be contributing to the disease, or if it is simply an opportunistic.

Overall, this is an interesting study that will improve the knowledge about modifications observed in swine dysentery diseased pigs.

**Specific comments:**

Line 88: As diet is considered an important variable to the development of swine dysentery, it will be interesting to add supplementary table with the diet composition including ingredients and protein, energy, macro and microelements.

A: We have added the reference for the diet used in line 89 and the diet composition in a supplementary table 1 (S1_File).

Reference 31 is not cited in the text. Please include this citation or delete it from the list of references.

A: Acknowledged. Reference 31 (updated for 32) included (Line 151).

Line 244: SCFAs is cited for the first time as an abbreviation. What does it mean?

A: Thank you, we have addressed this in lines 254 and 255.

Line 272: “….findings, Burrough et al. (11) found…”

A: Acknowledged. Line 283.

**Reviewer #3:** The manuscript deals with a relevant and interesting theme. This topic will be of interest to the readership of PLOSONE.
Generally, the structure of each section of the paper is very clear and well defined.

The methods used are sound and properly described.
The results are interesting and well discussed.
The limits of the study are reported within the text with a sufficient discussion.
The English is correct and fluent for the readers.

Not all the images included in the paper are sharp enough. Authors should submit better quality figures.

A: Acknowledged. Figures have been updated to publication quality.
I did not find the captions of figures, authors should provide them.

A: PLOS formatting guidelines is different from most journals. Captions are included in the text as figures are cited, instead of at the end of the manuscript. Figure 1 is in L167, Figure 2 L180, Figure 3 L186, and Figure 4 in L196.

Lines 263 and 266: please modify “actinobacteria” in “Actinobacteria”
A: Acknowledged (L274 and L277).

Line 287: ante-mortem should be italicized
A: Acknowledged (L298).
